# Patient safety incidents in anaesthesia: a qualitative study of trainee experience from a single UK healthcare region*

**DOI:** 10.1111/anae.16462

**Published:** 2024-11-03

**Authors:** Amelia Robinson, Amanda Kelsey, Sara McDouall, Helen Higham

**Affiliations:** ^1^ Nuffield Department of Anaesthesia Oxford University Hospitals NHS Foundation Trust Oxford UK; ^2^ School of Healthcare University of Leeds Leeds UK; ^3^ Department of Anaesthesia Royal Berkshire Hospital Reading UK; ^4^ Thames Valley School of Anaesthesia and Intensive Care Medicine UK; ^5^ University of Oxford Oxford UK

**Keywords:** debriefing, patient safety incidents, qualitative methodology, training

## Abstract

**Background:**

Anaesthetic training has always had patient safety as part of the curriculum. However, there is limited emphasis on what happens when things do not go to plan. Our aims were to understand the impact of involvement in patient safety incidents on anaesthetic trainees in our region, to describe the range of support currently offered and put forward suggestions for improvement.

**Methods:**

An initial electronic survey was sent to all anaesthetic trainees in a single UK healthcare region to capture qualitative and quantitative information on patient safety incidents. After completing the questionnaire, participants were asked to consent to involvement in a semi‐structured interview to provide a more detailed understanding of the impact of safety incidents. Data were analysed from the questionnaires and interview transcripts using descriptive statistics and thematic analysis.

**Results:**

Thirty‐four completed questionnaires were analysed revealing 27 trainees had been involved in a patient safety incident. Ten semi‐structured interviews were conducted and six themes were identified: team dynamics (including adequacy of staffing and supportive departmental culture); context of the event; reflex immediate support post‐event; working environment pending completion of the investigation; personal impact (including physical and mental health); and suggestions for future support.

**Conclusion:**

This study has shown the significant impact of safety incidents on anaesthetic trainees in one training region in the UK and highlights the importance of implementing early, tailored debriefs led by trained facilitators, the value of a supportive work environment and the need to raise awareness of system‐based approaches to learning from incident investigations. Further research should guide the format and delivery of support for trainees to provide more helpful and timely interventions after patient safety incidents and reduce the risk of future harm to both patients and trainees.

## Introduction

A patient safety incident is defined by the General Medical Council as “*any unintended or unexpected event which could have or did lead to harm of one or more patients*” [1]. The publication of the Patient Safety Incident Response Framework from NHS England [2] places emphasis on systems‐based rather than person‐centred approaches, with learning from patient safety incidents highlighted as the most important aspect of the investigation. This “*prioritises compassionate engagement with those affected by patient safety incidents*” and as a result should contribute to the growth of a just culture [3]. Given this new framework, it is important to know how anaesthetic trainees currently experience patient safety incidents.

Over the course of a career, most anaesthetists will experience at least one peri‐operative safety event [4]. These events are known to have a significant impact on the wellbeing of staff involved, which has been identified as the ‘second victim’ effect [5]. Furthermore, there may be ongoing effects on patient safety for healthcare professionals who are involved in a patient safety incident [6]. A search for relevant previous literature found 10 studies which examined the reaction to patient safety incidents among anaesthetists [7, 8, 9, 10, 11, 12, 13, 14, 15, 16]. Only three were UK‐based [9, 10, 14] and none of these focused on anaesthetic trainees.

Trainees may be more susceptible to damage to their clinical confidence [17]; three non‐UK‐based studies (from the USA, Australia and South Africa) that studied the impact of involvement in patient safety incidents on trainees confirmed this [7, 11, 12]. Arriaga et al. found that a communication breakdown during a patient safety incident led to a decreased likelihood of a proximal debrief following the event [7]. Tan et al. found that a proximal debrief, if delivered appropriately, is valued by anaesthetic trainees [11]. Jithoo et al. commented on the blind spot in anaesthetic training in terms of how to handle the aftermath of a peri‐operative death, especially compared with other specialities, despite the inevitability of patient safety incidents when working in anaesthesia [12].

The aims of this study were: to understand the impact of involvement in patient safety incidents on trainees from a large, diverse region (Thames Valley); to explore the factors which protect from, or exacerbate, the development of the ‘second victim’ effect on anaesthetic trainees experiencing a patient safety incident; and to describe and evaluate the range of support offered to trainees in the aftermath of patient safety incidents.

## Methods

Ethical approval was sought from the clinical trials and research governance board at the University of Oxford. This study was classed as quality improvement work and subsequently the need for further formal ethical approval was waived. This report was prepared according to the Standards for Reporting Qualitative Research [18]. Written informed consent was taken from all participants following explanation of the purpose of the study for data recording and the dissemination of anonymised results.

Recruitment was via an email invitation to complete an anonymous preliminary questionnaire which was distributed to all anaesthetic trainees in the Thames Valley region (105 specialist trainees and 35 core trainees), with a reminder email sent 3 weeks later. After completion of the questionnaire, participants were offered the opportunity to take part in a further, voluntary individual semi‐structured interview [19]. We chose a qualitative research approach because it “*is most revealing when the variables of greatest concern are unclear*” [19], as we had limited knowledge (aside from personal experience) of what the key problems would be. Interviews were undertaken to provide a richer understanding of trainee experience and because it is recognised that questionnaire responses can reflect what people think they should say, rather than actual personal beliefs or experiences [19].

The initial questionnaire and interview structure (online Supporting Information Appendices S1 and S2, respectively) were written with reference to the existing literature on the topic which used surveys [8, 9, 10, 11, 13, 20] and semi‐structured interviews [7, 12, 21]. Development of an interview structure was important to ensure uniformity, while allowing new ideas to be shared and explored [22]. The survey and interview guide were pilot tested, discussed and optimised with clinical psychologists working within the local Integrated Care System. During this process, changes were made; for example, a list of sources of support for participants was added at the end of the survey. The potential to cause anxiety or distress during discussion of sensitive topics is a known hazard in healthcare qualitative research [23] and, for this reason, we chose individual interviews rather than focus groups.

All interviews were undertaken by the principal investigator (AR) for consistency and followed a standardised interview structure. The interviews took place using videoconferencing software to conform with the social distancing guidance in force at the time, due to the COVID‐19 pandemic. Responses were confirmed with interviewees as the interview progressed and at the end.

The interviews were audio transcribed using voice recognition software (Otter.ai, version 3.8.0.772, Mountain View, CA, USA) and the transcripts were manually checked against the recordings for accuracy and any identifiable personal information was removed. Qualitative analysis began after three interviews to facilitate the assessment of thematic saturation [24]. The transcripts were analysed independently by AR and AK using thematic analysis [25] to assign codes to pertinent common concepts between interviews (Table 1). By inductive reasoning, 28 categories were generated from the data and the investigators then discussed and iteratively reduced them until six unifying themes were agreed. Following agreement on the six themes, all transcripts were re‐reviewed by both analysts to ensure comprehensive interpretation. Thematic saturation was achieved when the analysis of three consecutive interviews provided no new information or themes for either analyst [24, 26].

**Table 1:** Illustrative example of the analytical stages of the interview process.

Full quote	Code	Condensate	Interpretation
It was just me and a couple of these nurses, and I wasn't quite sure what was happening. So, at this point, I was like, I still don't know why this has happened. And I was like when you're trying to work out what's going on and I was like, I wasn't sure what was happening. And then I admit, I possibly got task fixated because no one else can do the airway, and anyone can do chest compressions. So, I got the nurses to start the chest compressions but none of them could do the airway. I've got to do the airway, but then I felt like a bit out of control leading the arrest. There weren't any doctors there, it was just me and two senior nurses	Insufficient staffing	I wasn't quite sure what was happening… I possibly got task fixated because no one else can do the airway… I felt a bit out of control leading the arrest…There wasn't any (other) doctors there. It was just me and two senior nurses	Inadequate level of staffing increases feeling of being overwhelmed during the incident and potentially exacerbates the second victim effect

An important aspect of qualitative research is acknowledging the impact the research team may have on the data due to their own experiences [24]. In our study, AR is an anaesthetic trainee with relevant clinical experience. This may have encouraged the volunteers to be candid during the interviews as they were conducted between peers. In contrast, AK has a social science background with experience of qualitative research on healthcare topics but no personal familiarity or relevant clinical anaesthetic experience. This introduced a perspective from a non‐clinical viewpoint, as AK had no pre‐existing information about the participants or experiences described by them. To address reflexivity, AR considered and documented her own understanding before commencing data collection and continued to reflect throughout the process with the use of a study diary. This helped to distinguish the participant's experience and opinions from those that of the interviewer and mitigate the potential for bias due to AR's professional role.

## Results

Survey responses were received from 34 trainees, which represents a 25% response rate. The respondents varied in age, from 25 to 40 y, with the majority (17/34) aged 31–35 y, which reflects the demographics of the trainees in the region. There was a slightly higher proportion of female respondents (n = 18) than expected given there were less female than male trainees in the region. In total, 27 trainees reported involvement in a patient safety incident. The reported events spanned several years, with just under half occurring within the previous 2 years (n = 13) and three occurring more than 5 years previously. Seventeen incidents resulted in patient death or life‐altering injury.

Every event impacted on the professional lives of trainees, ranging from loss of confidence at work (n = 11) to consideration of a career change (n = 3). Impact was also experienced in their personal lives, for example, difficulty sleeping (n = 10). Following the event, most respondents (n = 18) had a ‘proximal debrief’ (i.e. a brief focused conversation with a senior colleague following the event) [27]. Most of those who had a proximal debrief found it helpful (n = 12). Five were offered a specific structured peer delivered review within 72 h of the event, which originated from the military and is known as TRiM (Trauma Risk Management) [28, 29]. However, eight received no immediate support following their incident. Most incidents (n = 24) had a subsequent investigation, with a quarter of these at an external or coronial level. Less than half of the respondents (n = 10) were informed of the ongoing investigation process, this was even though 13 had been asked to write a statement for the investigation. The majority of respondents (n = 24) had not received any training on how to write a witness statement. Most respondents (n = 24) felt that education on how incidents are investigated would be beneficial.

Subsequently, 14 of the respondents volunteered for individual semi‐structured interviews (conducted between 30 March and 5 April 2022). Thematic saturation occurred after the analysis of 10 interview transcripts. The 10 volunteers were aged between 32 and 39 y with an even male‐to‐female ratio. The median interview duration was 32 min (ranged 21–70 min). The interviewee demographics are described in Table 2 and the investigation process and support offered are shown in Table 3.

**Table 2:** Demographics of interview volunteers and details of patient safety incidents.

Interview number	Age; y	Sex	Number of years training (FTE)	Incident in or out of hours	Most senior anaesthetist present	Location	Time duration between interview and incident; months
1	32	Female	5	Out	Consultant	ICU	32
2	32	Female	2	In	Consultant	Labour ward	7
3	38	Female	5	Out	Solo	ICU	24
4	39	Male	9	Out	Registrar	Emergency department	60
5	32	Male	3.5	Out	Consultant	Emergency theatre	1
6	38	Female	8	Out	Solo	ICU	48
7	36	Male	3.5	Out	Solo	Car park	24
8	36	Female	7	Out	Solo	Emergency theatre	3
9	32	Male	4.5	Out	Solo	ICU	18
10	34	Male	4.5	Out	Consultant	ICU	32

FTE, Full‐time equivalent; ICU, intensive therapy unit; in hours, 07.30–18.00 Monday to Friday; out of hours, all other times.

**Table 3:** Support following the patient safety incident, level of investigation and duration of time trainee felt impacted.

Interview number	Immediate support	Time away from work following incident	Subsequent support	Level of investigation	Informed of outcome of investigation?	Duration of time felt affected by incident
1	Proximal team debrief following morning	No, but asked if they felt able to continue night shift	Left Trust 3 weeks later, no ongoing communication about investigation without prompting	Internal investigation	Not invited to morbidity/mortality meeting, received copy of investigation report months after distribution	5 months
2	Cup of tea with anaesthetic team immediately after event	No	None	Unaware of what investigation occurred	N/A	2 weeks, but for whole pregnancy following event
3	Coffee next morning with senior registrar	No, as felt had no choice but to go in for next night shift	None	None	N/A	Ongoing (more than 2 years)
4	Proximal team debrief later in the shift	None	Consultant handled investigation documentation, and helped trainee access occupational health	Referred to coroner	No	Ongoing (more than 6 years)
5	Proximal theatre team debrief, and then anaesthetic team debrief	None	TRiM	Referred to coroner	N/A (yet to occur at time of interview)	1 week
6	None	None	Limited support, even when approached a consultant directly for advice on statement writing. Educational supervisor away.	Coroner's inquest	Yes	Ongoing (more than 4 years)
7	Proximal team debrief at end of shift	None, but was offered time off	Regular check ins by educational supervisor and college tutor. Offered psychological support (around both incident and inquest)	Coroner's inquest	Yes	7 months
8	Discussed with peers	None	None	None	N/A	2 weeks
9	Proximal team debrief at end of shift	None	TRiM Discussion of clinical scenario with seniors and peers	Not aware of any	N/A	4 weeks
10	Proximal team debrief at end of shift	Completed shift, then 3 days sick leave	None	Internal	Not without prompting	Ongoing (more than 2 years)

TRiM, Trauma Risk Management; N/A, not applicable.

The six themes and subthemes identified are described below (Table 4). Our analysis identified positive and negative contributory factors which impacted on the trainees within each theme.

**Table 4:** Themes and subthemes which emerged from analysis of interviews with ten anaesthetic trainees in the Thames Valley region who had experienced a patient safety incident.

Team dynamics
Subthemes: ‐ Adequacy of staffing of team ‐ Familiarity of the trainee within team ‐ Impact of hierarchy on team efficacy ‐ Conflict impeding team communication
Context of the event
Subthemes: ‐ Degree of personal identification with patient and resultant impact on empathy felt ‐ Working at limit of competency as a trainee and volume of work outstanding ‐ Time of day and duration of event ‐ Availability of appropriate support
Reflex immediate support post event
Subthemes: ‐ Presence of experienced colleague available and prepared to offer support immediately following event ‐ Proximal debrief: timing and setting ‐ Proximal debrief: degree of support demonstrated by leader ‐ Expectation to continue working or take a break
Working environment pending completion of the investigation
Subthemes: ‐ Feeling of isolation ‐ Presence of gossip and individual blame ‐ Barriers to discussing details of event ‐ Communication about and support during, progress of investigation including involvement of the coroner ‐ Support enabling learning from event for future practice
Personal impact
Subthemes: ‐ Difficult negative emotion ‐ Impact on physical and mental health ‐ Threat to career or difficulty in training
Suggestions for future support
Subthemes ‐ Appropriate person and setting for support and care of the individual trainee ‐ Support from event to conclusion of investigation ‐ Pre‐emptive training on investigation process and statement writing ‐ Coroner's court unfamiliarity and gap in training

Within the team dynamics theme, we explored how the make‐up of the clinical team responding to the patient safety incident and the culture of the anaesthetic department affected the participant's experience of the event. Four subthemes emerged. First, adequacy of staffing, a factor which trainees are unable to control. However, an appropriate level of staffing of the team had a significant impact on how effectively they felt they were able to work during a patient safety incident, and how positively they felt reflecting back on their experience. Working within a well‐staffed and well‐functioning team was a positive factor but inadequate staffing was a negative factor. For one trainee, the inability to ensure working within an adequately staffed team felt like an uncomfortable transgression of how the system should function:

*“What was asked or expected of staff commonly felt beyond what we were capable of delivering, because of capacity and staffing…we will be doing our best in conditions that we wished were better… A lot of us have felt the tension between we're working within a system and feel part of and responsible for care that we're giving in a system, the adequacy of which feels outside our own control. And that's a really difficult tension to exist in when you also feel personally held accountable for outcomes.”* (Trainee 6)

Second, familiarity with the team; if trainees were familiar with the team and department this was protective. This helped to facilitate performance during the patient safety event, for example, knowing team members names allowing effective leadership during an emergency. Being familiar with the team also enabled a feeling of being a visible and valued member of a department who may need support following a patient safety incident, rather than a relatively unknown new member of staff. However, being a trainee and, therefore, a member of the rotational workforce does not necessarily lend itself to feeling incorporated and safe as part of a department:

*“I think that's one thing that is difficult is a sense of isolation and constantly moving as a trainee, you may or may not have a close colleague that you feel comfortable…that you feel you can confide in at the time.”* (Trainee 6)

Third, impact of hierarchy on team efficacy. Several trainees commented that, in their experience, the impact of a steep or engrained hierarchy impeded the resolution of their patient safety incident:

*“The ICU registrar said to the anaesthetic consultant you need to come out and bag and was ignored. And so, then he said I think we should set up for a surgical airway. And I can't remember exactly what words he used, but he was told not to do that. The registrar for the night came back with a video laryngoscope offered it to the consultant and was turned down again… she noted the saturations and said have you tried bagging the patient, she was ignored. And then she was the second person saying that we need to think about a surgical airway and was absolutely told no.”* (Trainee 10)

In order to complete training, there is a process of continuous assessment to reach the level required to practice as a consultant. Several participants felt that this perceived pressure to always perform well within a department was a barrier to accessing support following the patient safety incident. This barrier felt larger if they were in a minority group:

*“You've got that additional pressure of looking good because it's [place name] …there's no room for weakness…it's only for the best…you're not going to come across amazing. And then you've got the subtext of I'm not the right colour, I'm not the right class. So, I've got to come across as looking really good all the time…So I probably wouldn't have volunteered this stuff to some of these people anyway.”* (Trainee 3)

Finally, conflict impeding team communication. The presence of conflict within the team at the time of a patient safety incident can lead to impaired communication during the event and also inhibited learning post event:

*“I think the dynamic between paediatrics and anaesthetics was slightly difficult for this scenario. It was very much heated…it was a really awful situation…but then I think afterwards, there was no talk of a joint debrief or leaning.”* (Trainee 2)

With the context of event theme, we captured how the experience of a patient safety incident was influenced by factors such as the patient characteristics, clinical environment and time of day. Four subthemes emerged. First, degree of personal identification with patient and resultant impact on empathy. The same event might be experienced completely differently by two trainees because of professional and personal experience, and individual biases. Patient background was relevant to the degree of empathy felt by the trainees. A good patient outcome is less likely in patients who are critically unwell and, as a result, incidents that occurred when treating such patients were less negatively impactful for the trainees. Patient characteristics which negatively impacted health also diminished the impact of the poor outcome:

*“The patient was also unvaccinated…it may be helpful for us to cognitively label them as being: ‘Well, they died because they weren't vaccinated, not because of anything I did wrong’.”* (Trainee 9)

Individual resonance with patient characteristics also contributed to the feeling of personal identification. If the patient was a similar age or stage of life as the trainee, this influenced their response. Where the participants strongly identified similarities with the patients, they were more vulnerable to negative processing of the event:

*“Added to which my wife was seven months pregnant …it felt like some useless anaesthetist might do to her what happened with me…everything felt like it was that bit more real, and consequences were that bit more unavoidable and understandable.”* (Trainee 9)

Second, working at the limit of competency as a trainee and the volume of work outstanding. When the patient safety incident occurred at a time when the trainee was stretched to their limit of clinical competency or had a large amount of unfinished clinical work, their experience was more negative. This was more likely to occur when working out of normal working hours, which is compounded by the fact that it is more common to have a lack of appropriate support available when working night shifts:

*“This must have been about half past eleven (at night) …I got called over to this lady's bed space, her blood pressure was low, so I went over…but then after (the patient safety incident) …there was no break. I had to just carry on doing the ward round, because there's still eight patients left to see…the whole night was like that…really busy…then in the morning I was like this is just awful…this is the sort of stuff that makes you want to quit.”* (Trainee 3)

Third, the time of day and duration of the event. If a patient safety incident occurred at the end of a shift or outside of daytime working hours, then this exacerbated the second victim effect:

*“It was kind of a slow‐moving car crash over a period of hours, in a patient who was initially quite cardiovascularly stable…we did eventually progress into cardiac arrest…the patient died at four or five in the morning, which was pretty horrendous.”* (Trainee 9)

Finally, the availability of appropriate support. When the participants felt they had access to a supportive senior colleague they were more likely to process the incident well. If they felt as if there was no one to call on for support or if their senior colleagues were unsupportive, then the converse was true:

*“On a night shift …the consultant on call had gone home straight after the handover…often at this Trust the consultants would stay on the shop floor until 10:30 or 11:00 in the evening when it's busy just to kind of get control…but he came to the handover…jokingly said, ‘We don't have any beds, we have less than no beds. Good luck, off you go’. And he left.”* (Trainee 6)

With the reflex immediate support post‐event theme, we brought together the experiences of the participants directly following the patient safety incidents. The support offered reflexively by colleagues in the hours to days following the events varied in both quality and quantity. Four subthemes emerged. First, the presence of an experienced colleague available and prepared to offer support immediately following the event. Not all participants were offered a proximal debrief, but those that did receive a sensitive and discreet post‐event discussion with a senior colleague valued it highly. The seniority of the supporter was important to the participants. It was perceived that the more senior the supporter, the more likely the colleague would have had similar previous experience, and so would be able to empathise with the trainee. However, such discussions did not always take place. The lack of a suitable senior colleague to offer support was an isolating and distressing experience:

*“The consultant who was on call that night for the unit I had never worked with before, and he was retiring after that shift…. he had just completely evaporated and wasn't even contactable. He was certainly not a source of support …my own educational supervisor had already left overseas to go on their holidays.”* (Trainee 6)

Second, the timing and setting of the proximal debrief. Some trainees felt that unless they were free from clinical commitments, they were unable to focus fully on the support offered. In one example, this led to a delayed reaction for the trainee:

*“I was with a reg and a consultant who were both lovely and we had a quick cup of tea just after the event …I still had a bleep in my hand and at the back of my head, ‘Oh there's an epidural that's not quite working, I've got to fix that before handover’. So, it's only really until I got in the car that I thought oh my gosh, that's just horrible, it was awful, so I had a cry.”* (Trainee 2)

A particularly valued intervention consisted of being taken off the ward by a senior colleague at morning handover, who then delayed the ward round and spent half an hour discussing the incident with the trainee over coffee. This trainee commented that without their colleague's kind intervention, they probably would have quit their training. The privacy of the supportive discussion was also important for trainees and reflected the importance of ensuring a psychologically safe environment [20, 27] for these discussions. In one example, the trainee commented that 20 people were invited to talk about the event afterwards, and this was not a successful proximal debrief because of the lack of privacy.

Third, the degree of support shown by the leader of the proximal debrief. If the proximal debrief was used as an opportunity to assign individual blame for the event, then this was damaging for participants. Some trainees had a negative experience where they felt a senior colleague was undermining them, and this increased their negative experience of the patient safety incident:

*“He looked directly at me and said, ‘It would have been nice if we'd been able to record that so you could reflect on your bad practice’.”* (Trainee 10)

Finally, the expectation to continue working. The pressure to continue with work and not take time out for recovery was felt by several trainees. This was common if the incident occurred part way through a series of scheduled night shifts:

*“I had massive anxiety about getting back into work…but it was like I had no choice, I had to go in.”* (Trainee 3)

The working environment pending completion of the investigation theme was an important period for the trainees. This spanned the time after the event but before the conclusion of the investigation, which commonly took months to years. It was a common experience that this period was more difficult for the trainees than the patient safety incident itself, as they were living with uncertainty about the personal and professional implications of the completed investigation. This indicates that support is needed not just immediately following a patient safety incident but throughout the investigation process. Five subthemes emerged. First, a feeling of isolation; working within a department as a trainee without explicit support during an open investigation is isolating and damaging. The investigation process itself was part of the trauma for several trainees as they felt that until the investigation was complete, they may still be questioned about their competence:

*“This was almost two years after the incident… in the intervening time, the pervading experience was the sort of Sword of Damocles constantly over my head the entire time, which was very difficult to live through.”* (Trainee 6)

Second, the presence of gossip and individual blame. Informal chatter about the incident in the workplace at this time was distressing for those involved. As the information on the details of patient safety incidents is kept confidential, coffee room rumours can form based on imperfect information. This is difficult to manage as confidentiality should be maintained and therefore correction of rumours is difficult, even if you are witness to them circulating:

*“Everybody knew, so you'd be stood in the mess or in a corridor and somebody would be talking about it…there's that doctor…then they would tell a version of events that you'd be like, well none of that is true.”* (Trainee 7)

Third, barriers to discussing details of the event. Despite recent improvements in investigation methodology and approach, several trainees commented it felt like the investigation was looking for an individual to blame, rather than identifying system improvements to be made:

*“The individuals involved would be put through these very difficult investigations where it felt like there was an opportunity to blame somebody rather than change the systemic things that were wrong.”* (Trainee 6)

The person‐centred approach of the investigations and presence of gossip within the workplace added to the sense of isolation for the trainees. It was common that they felt inhibited from reporting or discussing details of the event, and several commented that they still do not feel confident to raise patient safety concerns as a result:

*“I find it really hard balance to strike of when to say something and when not to. And I feel like we should be in a system where we can say something in all these situations without having to worry what the outcome will be. But experience tells me that's not the case…there's still not a good system in place where individuals can feel protected to raise concerns.”* (Trainee 10)

The penultimate subtheme related to communication about, and support during, progress of the investigation, including involvement of the coroner. Due to the challenging atmosphere some participants experienced at work, trainees who did receive ongoing support throughout the investigation period valued it highly. Two trainees were given the option of referral to a TRiM service, which they valued:

*“Actually, just having a conversation with the normal, listening person who is really generous with their time, I found really helpful…it did close the loop quite nicely…it reflects a degree of success in that it's not lead to persistent nasty thoughts.”* (Trainee 9, referring to TRiM)

Given that this is a challenging time for trainees, it was surprising that there was a notable lack of support and poor quality communication during the investigation period for many. One trainee was asked for their recollections of an incident and to write them down, without any explanation of what this statement would be used for. In this example, emphasis was placed on the timeframe for administration of the investigation rather than on trainee support, resulting in them staying until the early hours of the morning to complete a statement:

*“When I was first asked to write things down about the incident, I was not told why or for what purpose, which I still think is just so entirely inappropriate… It felt like an underhanded way of getting you to write down without any of the safeguards you would expect.”* (Trainee 6)

Although trainees are familiar with writing medical notes, witness statements require a different approach; for example, rather than offering differential diagnoses, they should aim to avoid ambiguity and focus only on the description of the event rather than interpretation. As these statements are not often required, these skills are not well practised by trainees, and participants therefore appreciated practical support from more experienced colleagues. An example of helpful support was when one trainee was helped by their consultant to write a statement the next day, which they found valuable.

A particularly difficult aspect of the investigation process for those trainees involved was the coroner's court. Attendance at a coroner's court is a not frequent occurrence during training and there was a lack of experience of the coronial environment and processes among the participants, and a general feeling of unease. The communication around being called as a witness was distressing for the majority because they had not been prepared for it:

*“It was like basically a year of being told I would need to come to coroner's court as a witness…that was actually what I found stressful rather than the actual event…but then the night before I didn't have to go to court. It was a year of stress, for nothing*”. (Trainee 7)

As there is often a significant interval between conclusion of an investigation and a coroner's inquest, if there was poor communication on the progress of the investigation, a subsequent request for attendance at court can be a shock for trainees. In contrast, good quality communication about the investigation process and advice for coroner's court helped to allay anxieties:

*“Six months later, I was in fact out of the country at a conference when I got out of the blue an email saying…Her Majesty's coroner would like you to…I remember having a sort of out of body experience…completely dissociated…I felt instantly just very terrified…and any support or resources I got, I had to go looking for and found them quite difficult to access.”* (Trainee 6)

The final subtheme was support enabling learning from the event for future practice. All participants wanted to learn more from the patient safety incidents they had been involved in. However, if communication following the event was limited, this also impeded reflective learning and any subsequent improvements to clinical practice. Lack of open discussion with seniors was a barrier to trainees learning from the clinical picture:

*“It wasn't like someone sat down with me to talk about acute right ventricular failure, and how to manage it, which actually would have been quite helpful, still would be…if only to think about how I would manage this case in future.”* (Trainee 9)

Several trainees were either not informed about, or not able to attend at short notice, the morbidity and mortality meetings at which their patient safety incidents were discussed among the consultant body. This was disappointing for them, as morbidity and mortality meetings are valuable learning opportunities and may be a very useful context for trainees to learn from the incidents. Normalising facilitating attendance and sensitive discussion of patient safety incidents at departmental meetings is one option for improving the system and encouraging a positive patient safety culture. One trainee commented on how listening to consultants discuss difficult cases was an important role modelling learning event, normalising that even senior colleagues are affected by these events.

Another barrier to reflective learning was the lack of feedback with the result of the investigation. As in our survey, trainees reported that the absence of communication regarding the investigation conclusion was common. Several participants tried to seek out the investigation report from the incident that they had been involved in, with varying success in accessing the information. It was a common feeling among participants that consultants are kept better informed of investigation reports and post mortem results than trainees, even if they were the one directly caring for the patient. This unequal access to valuable information to improve clinical practice could add to a sense of isolation.

The patient safety incidents and subsequent investigations had significant impacts on participants. For many, the impact spread out from their professional into their personal lives. In the personal impact theme we sought to explore the influence on the physical and mental health of trainees and the duration of time they felt impacted. Three subthemes emerged. First, difficult negative emotions. Coming to terms with patient safety incidents was not just a professional matter. If support was not satisfactory, there was also often a significant impact on life outside work:

*“I was experiencing intrusive thoughts, flashbacks of certain elements, feeling that the world was a very unsafe place. Feeling very isolated, that it was difficult for other people to understand what it was I was going through.”* (Trainee 6)

The duration of the personal impact from the patient safety incidents was prolonged for some participants, and for a smaller proportion, they remained affected years later. The second subtheme was the impact on physical and mental health. One interviewee was diagnosed with post‐traumatic stress disorder. This individual commented it was difficult to manage as it felt like an ongoing, unresolved experience over the 2‐year investigation period:

*“It was the most stressful experience of my working life by a long way, with very significant effects on my mental, physical, emotional health…I was not sleeping well, lost quite a lot of weight, …found shifts stressful…because…I didn't feel necessarily very reassured that similar circumstances couldn't arise again…It was about four years ago, and I'm still affected by it.”* (Trainee 6)

Finally, threat to career or difficulty in training. The impact of these events was significant and for several participants led them to have thoughts of wanting to leave the profession. Even if there was no thought of leaving, the experience could cause the trainees to have doubts in their own practice:

*“I probably think about leaving every couple of months or so. You always question your own failings…Am I good enough to do this? What if it happened again? … What if you don't know what to do? Is someone going to die again?”* (Trainee 3)

Despite having a significant effect on the trainees, the actual impact on clinical judgement may not have been visible to others. This suggests ongoing sensitive support should be offered to all trainees, including those who appear to be coping exceptionally well:

*“All my clinical…work related reviews continue to be absolutely glowing…you wouldn't necessarily have known…I don't feel it impaired any of my ability to look after patients, but it was at a great cost to myself.”* (Trainee 6)

The final theme related to suggestions for future support. We brought together the participants' suggestions for improvement to the support offered following a patient safety incident and how small changes to the system could have a large impact on the experience of trainees. This included examples of when the support received by participants had helped them learn from the event and have the confidence to take positive learning into their future practice.

Most respondents had suggestions for how the risk of a negative impact could be mitigated. These focused on two main areas: a proximal debrief with a trusted senior colleague and subsequent ongoing support (which should be routine and not reliant on trainee request); and a preventative approach with routine teaching on the process of an incident investigation for all in early speciality training. Four subthemes emerged. First, the appropriate person and setting for support and care of the individual trainee. Participants valued highly the offer of a supportive proximal debrief and reported that it mitigated their negative ongoing experience of the event:

*“Just, ‘Do you want to talk about what happened?’ That would have been more than enough for me…if someone had just said that I probably wouldn't have left [training] …it's a really easy intervention that has a massive impact.”* (Trainee 3)

Several trainees also commented on the desire to discuss these events with fellow trainees in a safe environment as a form of peer support. Although this would not replace the lived experience of a senior consultant in post‐event support, talking with peers may facilitate a more relaxed and open discussion, and may serve to normalise the frequency of patient safety incidents among the trainee population.

The second subtheme was support from event to investigation conclusion. As this stressful period can be prolonged, participants commented they would value a senior colleague who would be available throughout this time for support:

*“I think the key is you need to have a good point of contact, if that is your ES [educational supervisor] who you get on with or someone else, it needs to be someone you can speak to fairly openly without reprisal…I'd want someone I know who had my back, rather than someone who was going to look at me like I'd done something terribly wrong.”* (Trainee 7)

There was a common feeling that trainees should not have to prompt the need for help themselves. A trainee who is feeling isolated during the investigation process may not be aware of support that is available, for example writing aids for witness statements.

The third subtheme was pre‐emptive training on investigation process and statement writing. A common idea among the participants was for proactive teaching on patient safety incidents. This would increase awareness that these events can occur during training and would act as a foundation for trainees so that their first experience of a patient safety incident is not such a surprise:

*“Softly introducing people to the fact that in anaesthetic training, they will almost certainly be involved in something quite nasty …putting out there early on and the ways to get support… ‘something [awful] has happened checklist’ of kind of the first 24 hours…almost like a blueprint of the things that you really have to do, So then the stress and the emotion of it, you don't have to worry about where do I turn first?”* (Trainee 10)

Finally, coroner's court unfamiliarity and gaps in training. Given the reported relative blind spot in training regarding how patient safety investigations are carried out (and particularly around coroner's inquests) specific training for coroner's court was valued greatly by trainees. A good example of support is described by one participant who benefitted from support before and after the coroner's inquest:

*“I think the coroner's side was more stressful for me than the actual event…my ES [educational supervisor] walked me through the coroner's stuff pretty usefully…the college tutor was very good about it too. So they were going to come with me, actually to the coroner's case, just to sit in the background and be there, which was really nice…they sent the result of the coroner's inquest round and offered a debrief afterwards…because I didn't go in the end it was quite useful to see what the coroner had said.”* (Trainee 7)

## Discussion

The overarching message from this study is that anaesthetic patient safety incidents can have significant professional and personal impacts on the trainees involved. This experience often extends until, and sometimes beyond, the conclusion of the investigation. It is not immediately obvious from the description of a patient safety event to what extent it will impact on a trainee involved, as it depends on the circumstances of the patient and incident as well as educational and institutional aspects of the working environment. With appropriate support tailored to the individual trainee from senior colleagues, departments and other sources, the second victim effect can be moderated. As far as we are aware, our study is the first to report on UK anaesthetic trainees' experiences of a patient safety incident. We hope to raise awareness of the potentially damaging impact these can have and to suggest what might be done to improve support.

The semi‐structured interviews revealed positive and negative factors affecting trainee experience. Knowledge of these will allow supervisors and departments to understand which events are more likely to adversely affect any individual trainee. Positive factors included working with a good level of staffing and being well‐established within a department. This replicated findings from studies in other countries. Arriaga et al. found that challenging team dynamics was a barrier to having a proximal debrief [7]. Tan found trainees who received debriefs were more likely to report feeling supported by their department [11]. Negative factors included identifying personally with the patient, for example in terms of a shared age or stage of life, as opposed to a patient who was critically ill and more likely to have a poor outcome. Jithoo et al. had a similar finding that patients peri‐operative status play a significant role in trainees' reaction to their death, and that if trainees interacted with the patient's family, they found it disturbing, perhaps because of closer personal identification with the patient [12].

This understanding of positive and negative factors could be used to undertake a rapid appraisal soon after a patient safety incident, in order to help identify those trainees at a particular risk of the second victim effect. Other factors such as the type of patient safety incident and time of day would also need to be considered. Ideally, all trainees involved in a patient safety incident would be offered an immediate debrief and referral to a TRiM service, as proposed by Johnson et al. [30]. If resources are constrained, the rapid appraisal could be used to prioritise access to this. This would be consistent with the ethos of the new Patient Safety Incident Response Framework which “*prioritises compassionate engagement with those affected by health patient safety incidents*” [2].

The interviews also showed that the investigation process can have a greater impact than the incident itself, particularly if the process is protracted. Previous studies have noted that the investigation can be traumatic, but not more so than the incident itself [4, 21]. Our findings suggest that this may be a UK‐specific phenomenon, perhaps due to differences in the way such investigations are undertaken.

Such trauma may be preventable through education. However, Aitkenhead pointed out that such training was “*virtually non‐existent*” [31]. There has been a shift more recently; for example, the Patient Safety Incident Response Framework offers guidance and training for facilitators leading after‐action reviews, which it recommends carrying out following most patient safety incidents. To build on this, training on the process of patient safety incident investigation and good practice in proximal debriefing could be added to specialty anaesthetic training. Our interviewees pointed out this was also true of training for coroner's inquests.

Our results reveal a concerning level of variability in support for anaesthetic trainees who are involved in patient safety incidents with evidence of long‐term impacts for a substantial proportion of the participants. Our results show what support is valued by trainees following a patient safety incident, specifically explicit and structured support from a trusted senior colleague, both immediately and for as long as required, during the investigation process.

Scott et al. found there is a predictable reaction among healthcare providers following an adverse patient event, and that their recovery may be improved with appropriate support [21]. They described three possible trajectories: ‘thriving’, when an individual made something good come from their experience, for example becoming an advocate for patient safety initiatives; ‘surviving’, when the individual performs at the expected level but continues to be affected by the event; and ‘dropping out’, which involved “*changing professional role, leaving the profession or moving to a different practice location*”. Our study has provided important insights into anaesthetic trainees' reactions to patient safety incidents and whether the support offered encouraged ‘thriving’, rather than just ‘surviving’ or even ‘dropping out’. It seems that the existing mechanisms for support of trainees are not always being activated appropriately, for example, notification to the regional Dean when any trainee is involved in a significant patient safety incident such as a Never Event [32] or an event requiring a patient safety incident investigation [2].

The optimal way to support staff following these events often requires an individualised approach and cannot be standardised. While most trainees valued a proximal debrief, as recommended in the literature [7, 11, 12], this was dependant on the setting, timing and supportiveness of the person leading the discussion. The proximal debrief was particularly effective if undertaken with a senior experienced colleague who could empathise, in a private location, when free from clinical commitments. Guidance for anaesthetists on how to handle this is offered in the literature [31, 33, 34]. The interviews showed that trusted colleagues are a highly valued source of support, which reflects the literature describing the importance of peer support [20].

**Figure 1:**
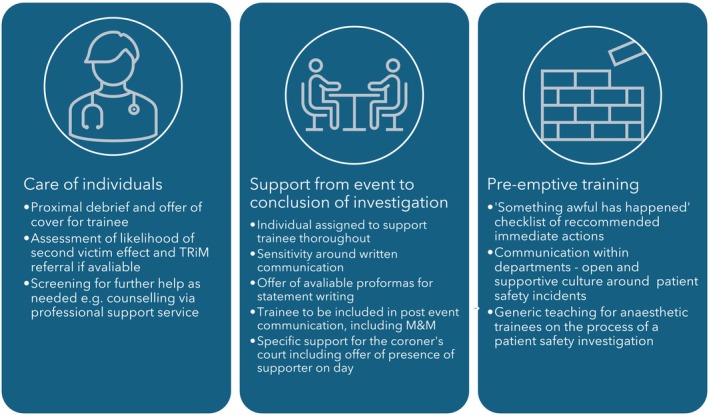
Recommendations for support of anaesthetic trainees in the aftermath of a safety incident. TRiM, Trauma Risk Management; M & M, morbidity and mortality meeting.

Participants in this study made several suggestions for how to improve future support for trainees involved in a patient safety incident. We have brought together these suggestions with some of those from the existing literature in Fig. 1. First, care of the individual includes the immediate actions to support a trainee following an incident, as in the ‘Team Immediate Meet’ tool [30], as well as measures that the interview participants found useful such as referral to TRiM [28]. Second, support from event to conclusion of investigation highlights that the investigation itself was part of the trauma for many of the participants. This covers the automatic offer of available resources, such as statement proformas, as well as a considerate communication style that the participants reported was lacking in some of the written communication (e.g. summons to the coroner's court). Finally, pre‐emptive training includes ideas from the participants on preparing all anaesthetic trainees for involvement in a critical incident.

A strength of our study is the use of qualitative methodology as it provided depth and richness to the analysis of trainee experience and ideas. The principal investigator was a fellow trainee, and therefore understood the context of working as an anaesthetic trainee and was perhaps perceived as less likely to be judgemental than a more senior colleague. This study had several limitations. There may have been selection bias. Respondents may have been more motivated to take part if they had suffered a particularly drawn out or negative experience. This is consistent with a relatively high proportion of the patient safety incidents reported in the survey resulting in external or coronial investigation. However, it should be noted that some trainees reported a positive experience. All the respondents were from a single Deanery and the sample size was relatively small. It may be that the quality of support offered to trainees differs elsewhere in the UK. However, the Thames Valley is a diverse region and is likely to be representative of trainees in the rest of the country. It is supportive that our results are consistent with findings in the international literature. The response rate to the survey was relatively low at 25%, but not significantly outside the normal range of online survey response rates [35]. A low response rate has the potential to introduce bias if there were a significant number of trainees with substantially different experience of critical incidents whose data were not captured. However, given the sensitivity of the subject matter, there were likely barriers for some trainees to respond, even to our anonymised survey. All of the semi‐structured interviews were carried out by a single interviewer. Although this ensured consistency and may have facilitated openness, this did potentially introduce bias as the interviewer could have emphasised specific themes they wished to explore. Finally, there is no objective measurement of the impact on retention within the specialty or sickness absence. In a future study these would be useful data to triangulate with qualitative findings.

In the current post‐pandemic climate, where reports on the state of education and practice are revealing the highest levels of stress and burnout among doctors in training since records began [36, 37], this study reveals that involvement in patient safety incidents adds to the burden. We have highlighted the importance of implementing early, tailored debriefs led by trained facilitators; the value of a supportive work environment; and the need to raise awareness of system‐based approaches to learning from incident investigations to remove the focus from individuals. Our profession can be justly proud of its focus on patient safety, but these results reveal a gap in the provision of training and support after patient safety incidents for anaesthetists at often vulnerable stages in their careers. Further research to guide the format and delivery of this support is likely to result in more helpful and timely interventions after patient safety incidents and, most importantly, reduce the risk of harm to both patients and trainees. We cannot afford to ignore this issue and risk losing trainees, from anaesthesia or other specialities in the current challenging work environment.

## Supporting information


**Appendix S1.** Initial survey.
**Appendix S2.** Interview structure.
