# Supplementary material for: Patient safety incidents in anaesthesia: a qualitative study of trainee experience from a single UK healthcare region*
Source: Anaesthesia. 2024 Nov 3;80(1):59–73. doi: 10.1111/anae.16462 (PMC11617131; doi:10.1111/anae.16462)
Supplement: Supplementary file 1 — Appendix S1. Initial survey. Appendix S2. Interview structure. [file ANAE-80-59-s001.docx]

**Appendix S1** - Initial questionnaire

*Anonymous questionnaire to be conducted online. Survey link distributed via email with awareness raised at teaching days just beforehand. In information about the survey, confidentiality emphasised. The aim is to improve the process for trainee wellbeing, not to investigate circumstances of patient safety incidents. All questions are optional, and there will be no requirement to complete the survey even if it has been started.*

1. How many years of anaesthetic training have you completed?
2. Have you been involved in a patient safety incident during your anaesthetic training?

Yes / No

1. What was the nature of this incident?

Serious incident ^1^ / Never event / Near miss / Other (please specify)

(1: Unintended or unexpected incidents, which lead to harm for one or more patients receiving healthcare)

1. What was the patient outcome?

No harm / Mild injury / Life-altering injury ^2^ / Death / Prefer not to say

(2: Permanent injury that interferes with daily life e.g. brain injury, amputation, ongoing disability)

1. When did the incident happen?

This month / This year / Within last 2 years / Within last 5 years

1. How did this event affect you?

Not at all /

Difficulty concentrating or feeling dissociated from people and events /

Difficulty sleeping /

Intrusive thoughts about the event /

Unusually irritable or angry /

Heightened awareness of potential dangers /

Loss of confidence at work /

Loss of enjoyment of work /

Considered taking time out of training or LTFT /

Considered career change /

Other (please specify)

1. Following the event, did you have either of the following:
   1. A “proximal debrief” (a brief focused conversation about the event within 24 hours)
   2. A peer delivered review within 72 hours, i.e. Trauma Risk Management (TRiM)
   3. Other immediate support (please specify)
2. If a proximal debrief occurred, how useful did you find this in allowing you to process the event

(Scale of 1 extremely unhelpful - 2 unhelpful - 3 neutral - 4 helpful - 5 extremely helpful)

1. Following the event, was it discussed at a departmental M&M?
   1. Were you informed about the date of this?
   2. Were you able to attend the M&M?
   3. Do you think that this was an effective way of disseminating learning from the incident?

(Scale of 1 extremely ineffective - 2 ineffective - 3 neutral - 4 effective - 5 extremely effective)

1. Following the event, was there an investigation process?
   1. If so, was this: at a departmental level, trust level or external
   2. Were you informed of the planned investigation process?
   3. Were you informed of the investigation outcome?
   4. Were you informed on how this would affect your ARCP process?

(Yes, No, Not applicable)

1. Were you asked to write a statement?
   1. Was this for a trust investigation or for the coroner?
   2. Have you received training on how to do this?
2. Were you asked to reflect on the incident in your portfolio?
   1. How comfortable did you feel doing this?

(Scale of 1 extremely uncomfortable - 2 uncomfortable - 3 neutral - 4 comfortable - 5 extremely comfortable)

- 1. Have you received guidance or training in how to do this?

1. Were you asked to attend coroner’s court?
   1. How comfortable did you feel doing this?

(Scale of 1 extremely uncomfortable - 2 uncomfortable - 3 neutral - 4 comfortable - 5 extremely comfortable)

- 1. Have you received guidance or training in how to do this?

1. Following the event, did you access help or support from any of the following sources (please select all applicable):
   1. Partner / Family / Friends,
   2. Fellow trainees,
   3. Consultants in the department,
   4. Consultants outside of the department,
   5. Educational Supervisor,
   6. College Tutor,
   7. Director of Medical Education,
   8. Occupational Health,
   9. PSWS services,
   10. BMA services / Employee assistance program
   11. GP,
   12. Practitioner health,
   13. Other (please list)
2. Was there a guideline that was followed for support of the staff following the incident?
   1. Are you aware of a departmental or trust guideline for this?
   2. Are you aware of who to contact for mental health support?
   3. Are you aware of who to contact for adjustments to your work pattern if needed?
3. If training was offered on how incidents are investigated, and what to expect if involved in one, how useful would this be as a trainee?

(Scale of 1 extremely unhelpful - 2 unhelpful - 3 neutral - 4 helpful - 5 extremely helpful)

1. If training was offered to educational supervisors on how incidents are investigated, and what to expect if your trainee is involved in one, how useful do you think this would this be?

(Scale of 1 extremely unhelpful - 2 unhelpful - 3 neutral - 4 helpful - 5 extremely helpful)

1. Is there anything else you feel would have helped you following the incident to help support you, if so please describe?
2. Would you be prepared to give a confidential online video interview (lead by a fellow anaesthetic registrar – Amelia Robinson) to explore how to improve support for trainees following patient safety incidents?
3. If so, please give the best contact email address.
4. Any other comments?
5. Which of the following best describes you?

Female / Male / Prefer to self-describe / Prefer not to say

1. Age?

21-25 / 26-30 / 31-35 / 36-40

1. What is your ethnic group?

White: British; Irish; Any other White background

Asian: Indian; Pakistani; Bangladeshi; Chinese; Any other Asian background

Black: African; Caribbean; Any other Black background

Other: Arab; any other background (please specify)

Mixed: White and Black Caribbean; White and Black African; White and Asian; Any other mixed / multiple ethnic background

We recognise that this questionnaire requires you to reflect on your experience of a patient safety incident at work, which may be distressing for you to think about. If you feel like you need support to deal with the impact of these events, we would encourage you to contact the following services: You can call your GP, or alternatively, access your local Staff Mental Health and Wellbeing Hubs which offer assessment, wellbeing advice, and fast-tracked referrals to further support if necessary. If you live and/or work in Berkshire, contact Wellbeing Matters on [wellbeingline@berkshire.nhs.uk](mailto:wellbeingline@berkshire.nhs.uk). If you live and/or work in Oxfordshire or Buckinghamshire, contact You Matter on [youmatter@oxfordhealth.nhs.uk](mailto:youmatter@oxfordhealth.nhs.uk). If you need urgent support, please call NHS 111, the Samaritans 116123, or 999 in an emergency

**Appendix S2** - Interview structure

1. Demographics:

Age

What gender do you identify as?

Ethnicity

Years of anaesthetic training completed

1. Event details:

Brief description of patient safety incident and the outcome for the patient (*If more than one event: which one would you like to focus on?)*

How did you respond to this event?

What impact did the event have on you: Professionally? Personally?

How long ago did it occur? For what time period did you feel affected by it?

Is this the first time this has happened? If something similar previously, how does it compare?

What do you think modified the impact of this particular incident?

*Add if needed: Were there any external factors e.g. culture, team, life events.*

What was the investigation process?

What communication did you receive about the investigation process? How did you experience receiving this communication?

1. Focus on the trainees needs immediately after the event both personally and professionally:

Following the event, what support did you receive? Specifically, for you personally? And for your professional development?

Were there any particular needs that were not addressed? What would have helped for you to feel supported or would have helped you manage better?

Was there an impact on your training and ARCP process?

Were there any external stressors for you at the time? Either professionally or personally? *If needed, give examples: Exam pressure*, *Pressure to achieve training competencies,* *Pressure from upcoming ARCP*; *Illness*, *Pregnancy / Becoming a parent*, *Bereavement*, *Difficulties in relationship or family issues*, *Financial pressure*, *Housing issues)*

Do you think these contributed to your response?

1. Stress management:

When you are stressed about something at work, how do you typically manage this?

Who do you typically turn to when you need advice or support about a work-related issue?

In your training how have you learnt to respond to adverse patient events? How specifically on a professional basis? And on a personal basis?

What is the best place or approach for anaesthetic trainees to learn about how to handle adverse events?

1. Support structures:
   - - - Based on this experience, what would you do differently if you were supporting a colleague going through the same thing?
       - What would be the ideal support or guidance to provide for a trainee following a serious incident?
       - Anything else you would like to share or comments about your experience?

Thank you for your time. If this has brought up anything which you would like support with, please consider contacting: your GP, or alternatively your local Staff Mental Health and Wellbeing Hubs

(If you live and/or work in Berkshire, contact Wellbeing Matters on wellbeingline@berkshire.nhs.uk. If you live and/or work in Oxfordshire or Buckinghamshire, contact You Matter on youmatter@oxfordhealth.nhs.uk.)
